# Prediction of ICU Patients’ Deterioration Using Machine Learning Techniques

**DOI:** 10.7759/cureus.38659

**Published:** 2023-05-07

**Authors:** Mohammed D Aldhoayan, Yosra Aljubran

**Affiliations:** 1 Health Affairs, King Abdulaziz Medical City, Riyadh, SAU; 2 Health Informatics, King Saud Bin Abdulaziz University for Health Sciences, Riyadh, SAU; 3 Clinical Research, King Fahad Medical City, Riyadh, SAU

**Keywords:** logistic regression, icu, learning algorithm, data mining, big data analytics

## Abstract

Introduction: Assessing vital sign measurements within hospital settings presents a valuable opportunity for data analysis and knowledge extraction. By generating adaptable, personalized prediction models of patient vital signs, these models can yield clinically relevant insights not achievable through population-based models. This study aims to compare several statistical forecasting models to determine their real-life applicability.

Objectives: The primary objectives of this paper are to evaluate whether the following measurements: blood pressure, oxygen saturation, temperature and heart rate can predict deterioration in Intensive Care Unit (ICU) patients. Additionally, we aim to identify which of these measurements contributes most significantly to our prediction. Lastly, we seek to determine the most accurate data mining technique for real-life data applications.

Methods: This retrospective chart review study utilized data from patients admitted to the ICU at a tertiary hospital between January and December, 2019. Data mining techniques for prediction included logistic regression, support vector machine classifier, k-nearest neighbors (KNN), gradient boosting classifier, and Naive Bayes classifier. A comprehensive comparison of these techniques was performed, focusing on accuracy, precision, recall, and F-measure.

Results: To achieve the research objectives, the SelectKBest class was applied to extract the most contributory features for prediction. Blood pressure ranked first with a score of 9.98, followed by respiratory rate, temperature, and heart rate. Analysis of 653 patient records indicated that 129 patients expired, while 542 patients were discharged either to their homes or other facilities. Among the five training models, two demonstrated the highest accuracy in predicting patient deterioration or survival at 88.83% and 84.72%, respectively. The gradient boosting classifier accurately predicted 115 out of 129 expired patients, while the KNN correctly predicted 109 out of 129 expired patients.

Conclusion: Machine learning has the potential to enhance clinical deterioration prediction compared to traditional methods. This allows healthcare professionals to implement preventative measures and improve patients' quality of life, ultimately increasing average life expectancy. Although our research focused exclusively on ICU patients, data mining techniques can be applied in various contexts both within and outside the hospital setting.

## Introduction

The ability to predict patient deterioration in intensive care units (ICUs) is vital for implementing timely interventions and optimal treatment strategies. This study aims to explore the feasibility of using data mining techniques to develop a prediction model for ICU patient deterioration based on vital sign measurements, contributing to the advancement of clinical decision-making and improved patient outcomes. Throughout their lives, individuals may experience various health conditions necessitating hospitalization for proper care. In certain cases, deteriorating conditions require patients to be admitted to the ICU for close monitoring and specialized treatment [1]. ICUs accommodate critically ill patients not only from general wards but also from emergency departments and post-operative care [2]. Given the critical nature of ICU patients, they require more attention and monitoring than those in other departments to detect potential deterioration or life-threatening changes [3].

The healthcare sector is transitioning from traditional practices to modern evidence-based care, primarily driven by data collected from sources such as electronic health records (EHRs) and monitoring devices [4]. Data mining explores large datasets to uncover valid, novel, and potentially useful patterns and relationships. In healthcare, data mining offers numerous benefits, such as disease-cause detection, risk factor analysis, length of stay prediction, and patient categorization [5]. One key application of data mining is clinical decision-making, which aids in diagnosis and treatment selection [5]. Monitoring systems in ICUs generate vast, complex, and unstructured data, often referred to as "big data" due to its size and processing challenges [6]. By leveraging intelligent technologies like big data analytics and decision support systems, healthcare professionals can identify patients at a higher risk of mortality, leading to better ICU decisions and improved clinical decision-making [7].

Previous studies have used data mining strategies to provide early deterioration alerts for ICU patients. For example, Mao et al. developed an integrated data-mining approach using time-series features and various data-mining techniques [8], while Nuaimi et al. focused on medical laboratory testing and feature selection to reduce dataset size [4]. In contrast, Liu et al. created a search engine for health-related information and discovered that summarized cluster-based predictions outperformed non-summarized data [9]. Additionally, Saeys et al. presented a taxonomy of feature-selection strategies and explored their potential in bioinformatics applications [10], while Cismondi et al. used artificial intelligence to predict future gastrointestinal bleeding lab test results in the ICU [11].

As the healthcare industry grapples with the challenges of analyzing vast amounts of data, big data has become a crucial driver of innovation and success. Data mining, the analytical stage of knowledge discovery, aims to extract valuable patterns or information from massive datasets [12]. Sampling and feature selection are essential aspects of dealing with large datasets, as they can help reduce the dataset's size while maintaining optimal model-building results [9,13]. This study aims to develop a prediction model for ICU patient deterioration, contributing to the growing body of research on data mining applications in clinical decision-making and providing healthcare professionals with valuable insights for improving patient care.

## Materials and methods

This study employed a retrospective chart review methodology to investigate patients admitted to the ICU at a tertiary hospital, King Abdulaziz Medical City, Riyadh, Saudi Arabia, between January and December of 2019. Relevant variables including diagnosis, medication, lab results, vital signs, and nursing and physician progress notes were extracted from the data governance department, and data from patients who declined to participate in the research were excluded. The data did not contain any missing values. This study was performed after receiving the Institutional Review Board approval from King Abdullah International Medical Research Center (approval number: SP21R/336/06).

Study subjects

This study collected data for adult patients aged 18 years and above admitted to ICUs at King Abdulaziz Medical City in Riyadh between January 2019 and December 2019. Data were obtained from patients who were transferred to the ICU from either the emergency department or general wards after their condition deteriorated. The design of this study involved a retrospective chart review, where electronic medical records were utilized to extract the necessary data for patients who were admitted to the ICU.

Given that this study involves a retrospective chart review aimed at predicting the probability of patient deterioration using clinical measurements such as blood pressure, oxygen saturation, and heart rate, all records of patients admitted to the ICU during the specified period were included as the study's sample, then inclusion and exclusion criteria were applied to filter out patients that were not satisfying these criteria. Patients younger than 18 years or older than 100 years of age and those who declined to participate and provide their information for research purposes were excluded from the study.

Statistical analysis

The purpose of this analysis was to predict the deterioration of patients in the ICU to enable efficient and timely management. Data management and analysis were performed using Python version 3.9 (Released 2020; Python Software Foundation, Wilmington, Delaware, United States). Data mining techniques, including logistic regression, support vector machine classifier, k-nearest neighbors (KNN) (specifically, K-Neighbors Classifier), gradient boosting classifier (specifically, XGBoost), and Naive Bayes classifier, were used to predict the probability of patient deterioration. The performance of these techniques was evaluated based on accuracy, precision, recall, and F-measure through a comprehensive comparison. All of the training and testing was performed by splitting the data into 80%-20% ratio for training and testing, respectively.

## Results

The analysis conducted involved the examination of 653 patient records. During the first 24 hours of admission, several dynamic monitoring data of physiological indicators were recorded for each patient, including six basic patient features (temperature, blood pressure, heart rate, respiratory rate, oxygen saturation, and body mass index). The dataset used in our research contains data from six different ICU departments: Surgical ICU, Neuro Critical Care ICU, Medical Cardiac ICU, Adult ICU Transplant & Oncology (AICU10), Adult ICU, and Adult Cardiac ICU. In total, there were 653 patients included in the dataset. The breakdown of patient numbers by ICU department is presented in Table 1.

**Table 1:** Distribution of patients across different ICUs in the hospital and the sample size available for analysis in each department

ICU Department	Patient counts
Surgical ICU	137
Neuro Critical Care ICU	136
Medical Cardiac ICU	146
Adult ICU Transplant & Oncology (AICU10)	77
Adult ICU	148
Adult Cardiac ICU	9

To achieve the research objective, the SelectKBest class was applied to extract the top best features that contributed the most to our prediction. Table 2 presents features that were obtained along with their respective scores and ranking.

**Table 2:** Ranking of top features

Feature	Score	Ranking
Diastolic Blood Pressure	9.980530	Rank-01
Systolic Blood Pressure	4.012045	Rank-02
Respiratory Rate	2.838670	Rank-03
Temperature	1.387034	Rank-04
Heart Rate	1.183335	Rank-05
Weight	0.725136	Rank-06
Height	0.542962	Rank-07
Oxygen saturation (SPO2)	0.107935	Rank-08
Body Mass Index	0.025374	Rank-09

For our prediction objective, 653 patient records were analyzed, out of which 129 patients had expired while 524 patients were still alive and discharged home or to another facility. Out of the five used training models, the gradient boosting classifier and K-Neighbors classifier had the highest accuracy in predicting patient deterioration or survival, with accuracy scores of 88.83% and 84.72%, respectively. The XGBoost classifier was able to correctly predict 115 patients out of the 129 expired patients, while the KNN classifier was able to correctly predict 109 patients out of the 129 expired patients. The results for each model are presented in Tables 3-7.

**Table 3:** Multinomial Naive Bayes classifier model accurately predicted patient status with an accuracy score of 66.67%

Class	precision	recall	f1-score
0	0.82	0.48	0.61
1	0.59	0.88	0.71

**Table 4:** Extreme gradient boosting (XGBoost) model predicted patient survival with an accuracy score of 88.34%

Class	precision	recall	f1-score
0	0.88	0.91	0.89
1	0.89	0.86	0.87

**Table 5:** K-nearest neighbors (K-Neighbors Classifier) model had an accuracy score of 84.72%, which means that the model predicted 109 out of the 129 deaths

Class	precision	recall	f1-score
0	0.86	0.85	0.86
1	0.83	0.84	0.84

**Table 6:** Support vector machine (SVM) classifier model predicted patient status correctly with an 80.56% accuracy score

Class	precision	recall	f1-score
0	0.87	0.75	0.81
1	0.75	0.87	0.81

**Table 7:** Logistic regression model predicted patient survival or expiration with an accuracy score of 75.93%

Class	precision	recall	f1-score
0	0.81	0.72	0.76
1	0.71	0.80	0.75

After comparing the five training models, we found that the gradient boosting classifier and KNN had the highest accuracy in predicting patient deterioration or survival, with accuracy scores of 88.83% and 84.72%, respectively. The GXBoost classifier was able to correctly predict 115 patients out of the 129 expired patients, while the K-Neighbors Classifier algorithm was able to correctly predict 109 patients out of the 129 expired patients. The accuracy scores for all models are presented in Table 8.

**Table 8:** Comparison of machine learning models' accuracy

Classifier Name	Accuracy Score
Naive Bayes Classifier	66.67
Gradient boosting classifier	88.43
K-nearest neighbors	84.72
Support vector classifier	80.56
Linear regression	75.93

## Discussion

Out of the total 653 patients included in our study, 129 patients were labeled as deceased while 524 patients were labeled as discharged and still alive. All patients were adults over the age of 18 and had spent more than 24 hours in the ICU. During the first 24 hours of admission, each patient's physiological indicators were monitored and recorded, including six basic patient features: temperature, blood pressure, heart rate, respiratory rate, oxygen saturation, and BMI.

Based on the results, we found that blood pressure had a significant relationship to patient deterioration with a Chi2 score of 9.98, followed by respiratory rate with a Chi2 score of 2.8. This is consistent with the findings of previous studies by Churpek et al. [14] and Kellett et al. [15], who also found respiratory rate to be an accurate predictor of clinical deterioration.

The results of this study demonstrate that a combination of vital signs and patient characteristics plays a crucial role in predicting ICU patient deterioration. It is evident that certain variables hold greater importance in the prediction model, with blood pressure parameters, respiratory rate, and temperature emerging as the most significant predictors. These findings align with existing literature, which highlights the importance of closely monitoring vital signs to detect early signs of patient deterioration in the ICU setting.

The ranking of the variables emphasizes the value of understanding the complex interplay between these factors, as they can collectively provide a comprehensive picture of a patient's overall health. The study reinforces the idea that no single variable should be considered in isolation when assessing ICU patients. Rather, a holistic approach that takes into account multiple factors is necessary to improve clinical decision-making and patient outcomes.

These results also underline the potential of data mining techniques in healthcare settings, particularly in the context of predicting ICU patient deterioration. By identifying the most critical features that contribute to the prediction model, healthcare professionals can prioritize these parameters when making clinical decisions and implementing interventions. This not only enhances the overall care provided but also contributes to the optimization of resources and targeted patient management.

To determine which machine learning models had the highest accuracy in predicting patient health status, we used five different prediction methods: logistic regression, support vector classifier, K-Neighbors Classifier, gradient boosting classifier, and Naive Bayes classifier. The top two accurate methods were gradient boosting classifier and K-Neighbors Classifier, with accuracies of 88.83% and 84.72%, respectively. The GXBoost classifier correctly predicted 115 out of the 129 expired patients, while the KNN classifier correctly predicted 109 out of the 129 expired patients.

To our knowledge, studies in the literature have yet to use these models in a similar dataset. Nuaimi et al. [4] used the same dataset as ours. However, they solely employed medical laboratory testing and relied on feature selection to lower the dataset size in their technique. In our study, we did not perform feature selection as we relied solely on the vital signs, which include only 10 features, and used all of them to accomplish the study objectives.

Limitations

It should be noted that this study was carried out at a single tertiary hospital in Riyadh, which may limit the generalizability of the results to other hospitals and healthcare systems. Additionally, machine learning techniques are highly dependent on the quality and standardization of the underlying data. The lack of standardization in the data could lead to significant discrepancies and impact the accuracy of the results. It is important to keep these limitations in mind when interpreting the findings of this study.

It is also important to note that the attributes used in the prediction were limited to the basic parameters used to monitor patient status. While these vital signs are important indicators of patient health, each patient admitted to the ICU has a wealth of hemodynamic information that could be used in future studies to improve prediction accuracy. Overall, while this study provides valuable insights into predicting patient deterioration using machine learning techniques, it is important to consider the limitations and potential areas for improvement in future studies.

## Conclusions

Machine learning techniques have the potential to significantly improve the prediction of clinical deterioration compared to traditional methods, allowing healthcare professionals to implement necessary prevention measures and improve patient quality of life. While this study focused solely on ICU patients, data mining techniques can be applied in various healthcare settings to improve patient outcomes. In this study, we used five different machine learning models and obtained interesting results, with two models revealing high accuracy in predicting patient survival or death. The identified factors, particularly diastolic blood pressure, systolic blood pressure, respiratory rate, temperature, and heart rate, are critical in predicting patient outcomes and should be prioritized in clinical decision-making.

We recommend expanding future research to include multiple ICUs in different hospitals and using standardized data to minimize discrepancies. Additionally, more attributes beyond the basic vital signs used in this study should be incorporated to improve prediction accuracy. Overall, the availability of big data in hospitals and other healthcare settings is an excellent opportunity to use machine learning techniques to improve patient outcomes and reduce costs. Additionally, longitudinal studies can help assess the model's performance over time and provide insights into the dynamic nature of ICU patient deterioration. Finally, developing machine learning algorithms to process large volumes of data can improve the model's generalizability and applicability in diverse clinical settings.
